# Proteostasis Response to Protein Misfolding in Controlled Hypertension

**DOI:** 10.3390/cells11101686

**Published:** 2022-05-19

**Authors:** Manuel Teixeira, Dário Trindade, Marisol Gouveia, Roberta Eller-Borges, Sandra Magalhães, Ana Duarte, Miriam Ferreira, Maria I. Simões, Maria Conceição, Alexandra Nunes, Ana Gabriela Henriques, Fernando Ribeiro, Sandra I. Vieira

**Affiliations:** 1 Department of Medical Sciences, Institute of Biomedicine—iBiMED, University of Aveiro, 3810-193 Aveiro, Portugal; manuelteixeira@ua.pt (M.T.); marisolgouveia@ua.pt (M.G.); robertaeller@ua.pt (R.E.-B.); sandra.vicencia@ua.pt (S.M.); alexandranunes@ua.pt (A.N.); aghenriques@ua.pt (A.G.H.); sivieira@ua.pt (S.I.V.); 2 Aveiro Institute of Materials—CICECO, University of Aveiro, 3810-193 Aveiro, Portugal; 3 Unidade Cuidados na Comunidade Cubo Mágico da Saúde, ACES Baixo Vouga, 3770-219 Oliveira do Bairro, Portugal; acduarte3@arscentro.min-saude.pt (A.D.); mzferreira@arscentro.min-saude.pt (M.F.); misimoes3@arscentro.min-saude.pt (M.I.S.); mcconceicao@arscentro.min-saude.pt (M.C.); 4 School of Health Sciences, Institute of Biomedicine—iBiMED, University of Aveiro, 3810-193 Aveiro, Portugal; fernando.ribeiro@ua.pt

**Keywords:** hypertension, protein aggregation, oligomers, fibrils, proteostasis, endothelin 1, ubiquitin, clusterin, body fluids

## Abstract

Hypertension is the most determinant risk factor for cardiovascular diseases. Early intervention and future therapies targeting hypertension mechanisms may improve the quality of life and clinical outcomes. Hypertension has a complex multifactorial aetiology and was recently associated with protein homeostasis (proteostasis). This work aimed to characterize proteostasis in easy-to-access plasma samples from 40 individuals, 20 with controlled hypertension and 20 age- and gender-matched normotensive individuals. Proteostasis was evaluated by quantifying the levels of protein aggregates through different techniques, including fluorescent probes, slot blot immunoassays and Fourier-transform infrared spectroscopy (FTIR). No significant between-group differences were observed in the absolute levels of various protein aggregates (Proteostat or Thioflavin T-stained aggregates; prefibrillar oligomers and fibrils) or total levels of proteostasis-related proteins (Ubiquitin and Clusterin). However, significant positive associations between Endothelin 1 and protein aggregation or proteostasis biomarkers (such as fibrils and ubiquitin) were only observed in the hypertension group. The same is true for the association between the proteins involved in quality control and protein aggregates. These results suggest that proteostasis mechanisms are actively engaged in hypertension as a coping mechanism to counteract its pathological effects in proteome stability, even when individuals are chronically medicated and presenting controlled blood pressure levels.

## 1. Introduction

Hypertension is a major modifiable risk factor for non-communicable diseases, particularly cardiovascular diseases (CVD), and one of the most prevalent disorders associated with ageing. It stands out as one of the most threatening complications for public health [1,2], not only because of the high mortality of CVD but also given the costs associated with hypertension treatment, especially in low-income countries [3,4]. Despite a decreasing tendency in global prevalence over the past decade, hypertension still affects approximately 20% of the population in high-income countries [1,5], particularly the elder individuals [2,6]. Persistently elevated blood pressure (BP) is the main feature of hypertension, a condition of multifactorial aetiology associated with both genetic and environmental factors [7]. Several systems act together to regulate BP and maintain physiological levels: the renin–angiotensin–aldosterone system, natriuretic peptides, the vascular endothelium, the sympathetic nervous system, and the immune system [8,9]. Among these, endothelial cells are responsible for the release of several vasoactive substances such as nitric oxide. This gaseous mediator promotes vascular smooth muscle relaxation to prevent elevated BP [10]. Vasoconstrictor molecules are also produced and released by endothelial cells, including Angiotensin II, Prostaglandin, or Endothelin 1, which act as a potent vasoconstrictor and are associated with endothelial dysfunction [11]. The balance between these molecules and other factors is crucial for regulating BP levels [8]. Disruption of this balance or malfunction of these regulatory systems generally leads to BP variability or increased BP that, when persistent, results in damage to cells, tissues, and organs, eventually leading to pathological conditions such as CVD [12,13,14].

Hypertension speeds up vascular ageing through alterations in several molecular pathways regarding endothelial function, oxidative stress, inflammatory processes, and potentially protein quality control [15,16]. In a mice model, hypertension was associated with a decline in protein homeostasis (proteostasis), resulting in a frail proteome with aberrant and misfolded proteins that are prone to aggregate [17]. Impaired proteostasis, the failure to accomplish protein quality control, is associated with the accumulation of several types of protein aggregates that might contribute to the pathophysiology of CVD-associated states such as idiopathic dilated cardiomyopathies [18], atherosclerosis [19], and heart failure [20].

Proteins are structurally dynamic molecules that can potentially acquire different conformational states throughout their lifespans. Additionally, proteins and peptides with low stability and high hydrophobicity, disordered regions, or natively unfolded molecules are prone to aggregate [21,22]. Protein aggregation is a dynamic and complex process involving a multitude of conformational intermediates, which drives misfolded proteins and peptides to form amorphous aggregates or regular aggregates, such as amyloid-like fibrils. During the aggregation process, early aggregates can undergo internal reorganization to yield more stable structures with β-sheets, such as prefibrillar oligomers. These species can then grow further by self-association or by interacting with other aggregates and forming highly structured fibrils [22]. Proteostasis is ensured by a dynamic and integrated network of protein quality control mechanisms that include: (i) molecular chaperones, responsible for proper protein folding, maintenance of the native conformation, and disaggregation [23]; and (ii) protein degradation systems such as the ubiquitin–proteasome system (UPS) and autophagy, which regulate protein abundance and disposal of unnecessary, damaged, or misfolded proteins [24]. Failure of the proteostasis network occurs when the cellular capacity to refold or degrade aberrant proteins is exceeded, leading to the accumulation of misfolded or damaged proteins and peptides that can aggregate and eventually engage in a multitude of deleterious interactions with a variety of cellular components [25].

The study of protein homeostasis in the cardiovascular system is mainly dependent on animal models. In fact, evidence from human subjects is scarce and generally limited to the analysis of tissues collected during very invasive procedures, such as endomyocardial biopsy, the gold standard to diagnose proteinopathies in cardiac disease [26,27]. Exploring protein quality in human plasma could unravel new diagnostic, prognostic, and therapeutic options with a simple, fast, and barely invasive procedure. Therefore, the main goal of this study was to characterize the protein aggregation profile of individuals diagnosed with primary arterial hypertension, its association with protein quality control mechanisms, and compare it with age- and gender-matched reference individuals without arterial hypertension.

## 2. Materials and Methods

### 2.1. Study Design

A total of 40 adults were recruited from a Primary Health Care Centre: 20 individuals clinically diagnosed with primary hypertension and 20 age- and gender-matched controls. To be included, patients with hypertension had to fulfil the criteria of essential hypertension provided by the ESH/ESC [28] and have been diagnosed for at least 6 months without changing their medication in the last 3 months before recruitment. Patients with secondary hypertension, evidence of target organ damage (i.e., heart, eye, kidney, and brain), coronary artery disease, heart failure, any previous cardiovascular event, peripheral artery disease, renal failure, or chronic obstructive pulmonary disease, were excluded. Ethical approval was guaranteed by the local Ethics Committee (Ref. 174211). All participants signed a written informed consent, and all procedures were conducted in accordance with the Declaration of Helsinki.

### 2.2. Clinical Data Collection and Sample Processing

Medical history, clinical data, medication, and demographic data were collected from clinical files and confirmed with the patients. Blood samples were collected by venepuncture of the antecubital vein into EDTA coated tubes, centrifuged at 2000× *g* for 15 min at 4 °C, and plasma divided into aliquots and stored at −80 °C until analysis.

### 2.3. Measuring Protein Aggregation with Fluorescent Probes

Proteostat Protein Aggregation Assay was purchased from Enzo Life Sciences (ENZ-51035, Farmingdale, NY, USA). Plasma proteins were incubated with the commercial reagent sensitive to protein aggregation at a ratio of 2 μL reagent to 98 μL of diluted plasma samples. Positive and negative controls were included, as indicated. Proteostat Protein Aggregation standards (ENZ-51039, Enzo Life Sciences, Farmingdale, NY, USA), containing stressed and unstressed IgGs, were used to establish a standard curve and calculate the percentage of aggregated protein in test samples. Fluorescence was measured using a Tecan Infinite M200 microplate reader (TECAN, Männedorf, Switzerland), with excitation set at 550 nm and an emission filter of 600 nm. For Thioflavin T (ThT) (211760050, Thermo Fischer Scientific, Waltham, MA, USA) staining, 5 µL of human plasma was loaded onto wells of a 96-well microplate, in duplicate. Samples were then mixed with 10 µM ThT in 100 mM sodium phosphate buffer, pH 7.4, to a final volume of 250 µL per well [29]. Fluorescence readings were acquired using the Tecan Infinite^®^ M200 PRO microplate reader at an excitation of 444 nm and emission of 485 nm.

### 2.4. Immunoassays

For slot blot analysis of immunotarget proteins and aggregated protein structures, plasma samples were transferred to nitrocellulose membranes using a Bio-Dot SF Microfiltration Apparatus (Bio-Rad Laboratories, Hercules, CA, USA). A total of 10 μg of each sample was loaded onto the spots and the vacuum was applied to transfer the samples to the membrane. Membranes were first reversibly stained with Ponceau S for loading control, and then blocked using 5% non-fat dry milk/1× TBS-T or 5% BSA/1× TBS-T for 2 h at RT. Primary antibodies rabbit polyclonal anti-amyloid fibrils OC (Merk KGaA), rabbit polyclonal anti-oligomer A11 antibody (Invitrogen, Thermo Fischer Scientific), mouse monoclonal anti-Endothelin 1 (NB300-526, Novus Biologicals, Littleton, CO, USA), and anti-ubiquitin (MMS-257P-20, BioLegend) were incubated overnight at 4 °C. Horseradish-peroxidase secondary antibodies (#7074S and #7076S, Cell Signalling) were incubated for 2 h and detected by enhanced chemiluminescence (ECL Select, Amersham, UK). Protein bands were detected with a ChemiDoc Imaging System (Bio-Rad) and analysed with Image Lab Software (Bio-Rad).

### 2.5. Fourier-Transform Infra-Red (FTIR) Spectroscopy

FTIR spectra were acquired using an ATR-FTIR spectrometer (Alpha Platinum ATR, Bruker, Billerica, MA, EUA), and pre-processed using OPUS software (Bruker, Billerica, MA, EUA). All spectra were recorded in the medium infrared region (4000–600 cm^−1^), with a resolution of 8 cm^−1^ and 64 co-added scans. A background spectrum was acquired with the crystal empty before each sample measurement. Each plasma sample (5 μL) was spotted into the crystal and air-dried prior to analysis in a room with controlled temperature (23 °C) and relative humidity (35%). Between each measurement, the crystal was cleaned with ethanol 70% and distilled water and dried to avoid cross-contamination and interferences in the spectra. Spectra were baseline corrected and normalized to amide I band. To resolve overlapping bands, a second-derivative analysis with a Savitzky–Golay algorithm and 3 smoothing points was performed. Principal Component Analysis (PCA) was applied to the 1700–1600 cm^−1^ spectral region, assigned to amide I band of proteins, to assess differences between groups related to protein secondary structures.

### 2.6. Statistical Analysis

Statistical analysis was performed with GraphPad Prism 8 software (GraphPad Software, Inc.). The D’Agostino–Pearson omnibus normality test was applied to test data distribution. Normally distributed variables were compared using the two-tailed unpaired t-test, while variables with an asymmetrical distribution were analysed using the non-parametric two-tailed Mann–Whitney test. Categorical variables were tested with χ^2^ test for between-group comparisons. The associations between two independent variables were tested using Pearson’s or Spearman’s correlations for normally distributed and non-parametric data, respectively. In the association studies, identified outliers were removed by the software. The level of statistical significance was set as α ≤ 0.05. For FTIR, all spectra processing and multivariate statistical analysis were performed using The Unscrambler X software v10.4 (Camo Analytics, Oslo, Norway). PCA was applied to the normalized second-derivative spectra for all samples in the amide I region.

## 3. Results

### 3.1. Participant’s Characteristics

The anthropometrics, BP, medical history, and medication of the participants are summarized in Table 1. There were no significant differences between groups in age, gender distribution, systolic and diastolic pressure, and heart rate. Body mass index and waist circumference were significantly higher in the hypertension (HT) group (Table 1). Some comorbidities and risk factors are shared between both groups (e.g., diabetes, dyslipidaemia, etc.), while obesity is more frequent amongst the HT group. The HT group had a very heterogeneous antihypertensive pharmacological distribution, but most of them were taking diuretics and Angiotensin II receptor blockers (ARB). A similar number of participants were taking statins in both groups.

### 3.2. Protein Aggregation in the Plasma of Individuals with Hypertension

**Figure 1:**
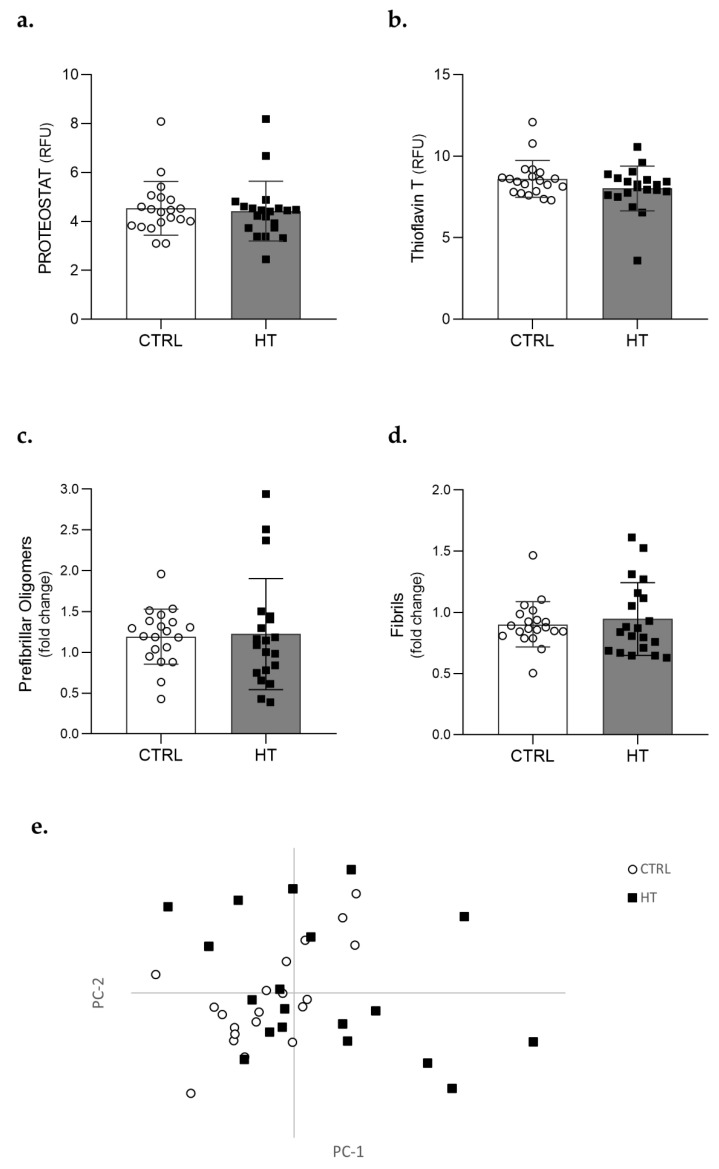
Protein aggregation profile in plasma samples of control and individuals with hypertension. Plasma protein aggregation was assessed by fluorometric assays with Proteostat (**a**) and Thioflavin T (**b**), immunoassays targeting conformationally distinct aggregates such as prefibrillar oligomers (**c**) and fibrils (**d**), and through FTIR (**e**). Two-tailed unpaired t-tests or Mann–Whitney tests were applied to compare controls (CTRL, ○) and individuals with hypertension (HT, ■) in fluorometric and immunoassays. All samples were normalized to the reference sample and levels are represented as fold changes relative to this reference sample. Bars represent mean ± S.E.M. FTIR data were obtained by Principal Component Analysis (PCA) applied to the normalized second-derivative spectra.

The effect of hypertension in plasmatic protein aggregation was initially evaluated using fluorescent probes, particularly the Proteostat^®^ dye which intercalates into cross-beta structures, and ThT, a benzothiazole dye known to bind misfolded and aggregated proteins. Analysis with either Proteostat or ThT showed that the overall amount of protein aggregates is relatively low in human plasma (at least in the conditions here tested), and no differences were found between the control (CTRL) and HT groups of individuals (*p* = 0.66 for Proteostat and *p* = 0.15 for ThT) (Figure 1a,b, respectively).

Considering the absence of significant differences in the amount of protein aggregates found in the plasma of HT and control individuals, we used conformation-specific and sequence independent antibodies to perform a structural evaluation of the plasma proteome. Results from these immunoassay analyses using conformation-specific antibodies showed no relevant differences in the amount of prefibrillar (*p* = 0.85) (Figure 1c) or fibrillar structures (*p* = 0.80) (Figure 1d) between both groups of individuals.

Structural evaluation of the plasma proteome was also performed using FTIR spectroscopy. FTIR spectra of all samples were analysed in the 1700–1600 cm^−1^ spectral region. Visually, all spectra looked identical, and a PCA analysis was applied to the second-derivative spectra in this region (Supplementary Figure S1). Results showed no discrimination between samples from controls and patients with hypertension, confirming that there were no significant differences in protein secondary structures (Figure 1e). Altogether, these data suggest that the protein aggregation profile is not altered in individuals diagnosed with and medicated for HT.

### 3.3. Proteostasis Biomarkers in the Plasma of HT Individuals

**Figure 2:**
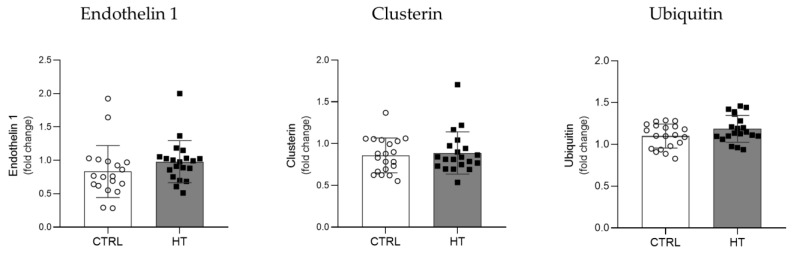
Levels of vasoconstrictor and proteostasis-related proteins in the plasma of control and individuals with hypertension. Plasma levels of Endothelin 1, Ubiquitin, and Clusterin were analysed by slot blot immunoassay. Two-tailed unpaired *t*-tests or Mann–Whitney tests were applied to compare both groups of individuals: controls (CTRL, ○) and individuals with hypertension (HT, ■). All samples were normalized to the reference sample and protein levels are represented as fold changes relative to this reference sample.

To assess the impact of hypertension on human plasma proteostasis, the levels of relevant biomarkers were evaluated by immunoblotting. Levels of Endothelin 1, a potent vasoconstrictor, were increased in HT samples compared to controls (*p* = 0.05) (Figure 2). Plasma levels of Clusterin, an extracellular chaperone involved in the proteostasis of secreted proteins, and Ubiquitin, a regulatory protein that selectively tags proteins for degradation, were also analysed. Both the HT and control groups exhibit identical levels of plasma Clusterin levels (*p* = 0.93) (Figure 2), while the total Ubiquitin levels were marginally increased in HT samples (*p* = 0.08) (Figure 2).

**Figure 3:**
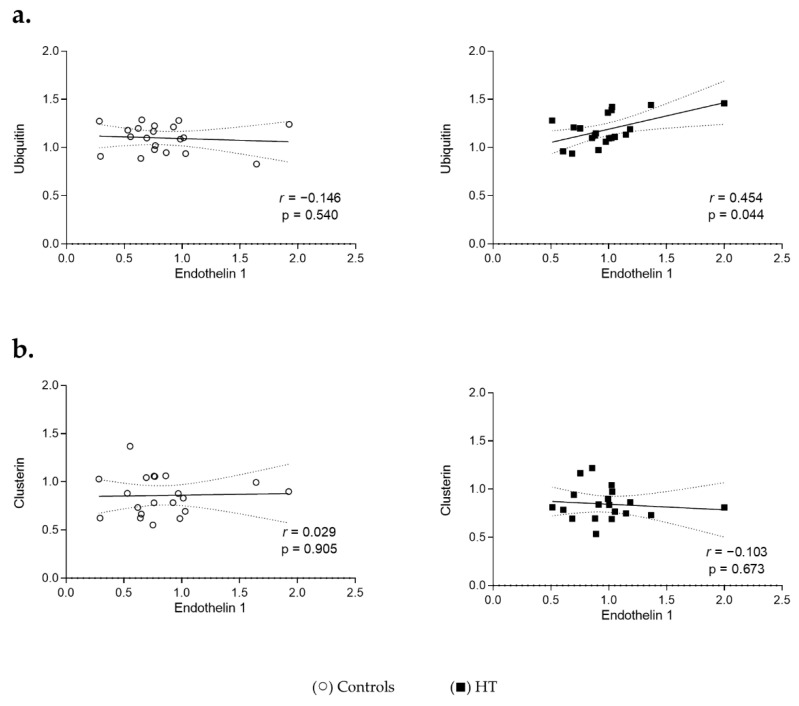
Association between hypertension and proteostasis-related proteins. The levels of Endothelin 1 in the plasma of controls (○) and individuals with hypertension (■) were matched with the corresponding individual levels of Ubiquitin (**a**) or Clusterin (**b**). Spearman’s rank coefficient was applied to test the strength and direction of the relationships between Endothelin 1 and Ubiquitin or Clusterin. The resulting coefficients (r) and corresponding *p*-values (p) are represented. *p* < 0.05 was considered statistically significant. All samples were normalized to the reference sample and protein levels are represented as fold changes relative to this reference sample. Lines represent a linear regression fit with corresponding 95% confidence bands (dotted lines).

Considering that high levels of Endothelin 1 lead to elevated BP, the association of this biomarker with the proteins involved in proteostasis was also evaluated (Figure 3). In the HT group, there was a positive and significant association between circulating levels of Endothelin 1 and Ubiquitin (r = 0.454, *p* = 0.04), which was not observed in control individuals (r = −0.146, *p* = 0.54) (Figure 3a). On the other hand, no association between Endothelin 1 and Clusterin was observed in either group (r = −0.103, *p* = 0.67 in HT group and r = 0.029, *p* = 0.91 in the CTRL group; Figure 3b).

### 3.4. Proteostasis and Protein Aggregation Associations in the Plasma of HT Individuals

**Figure 4:**
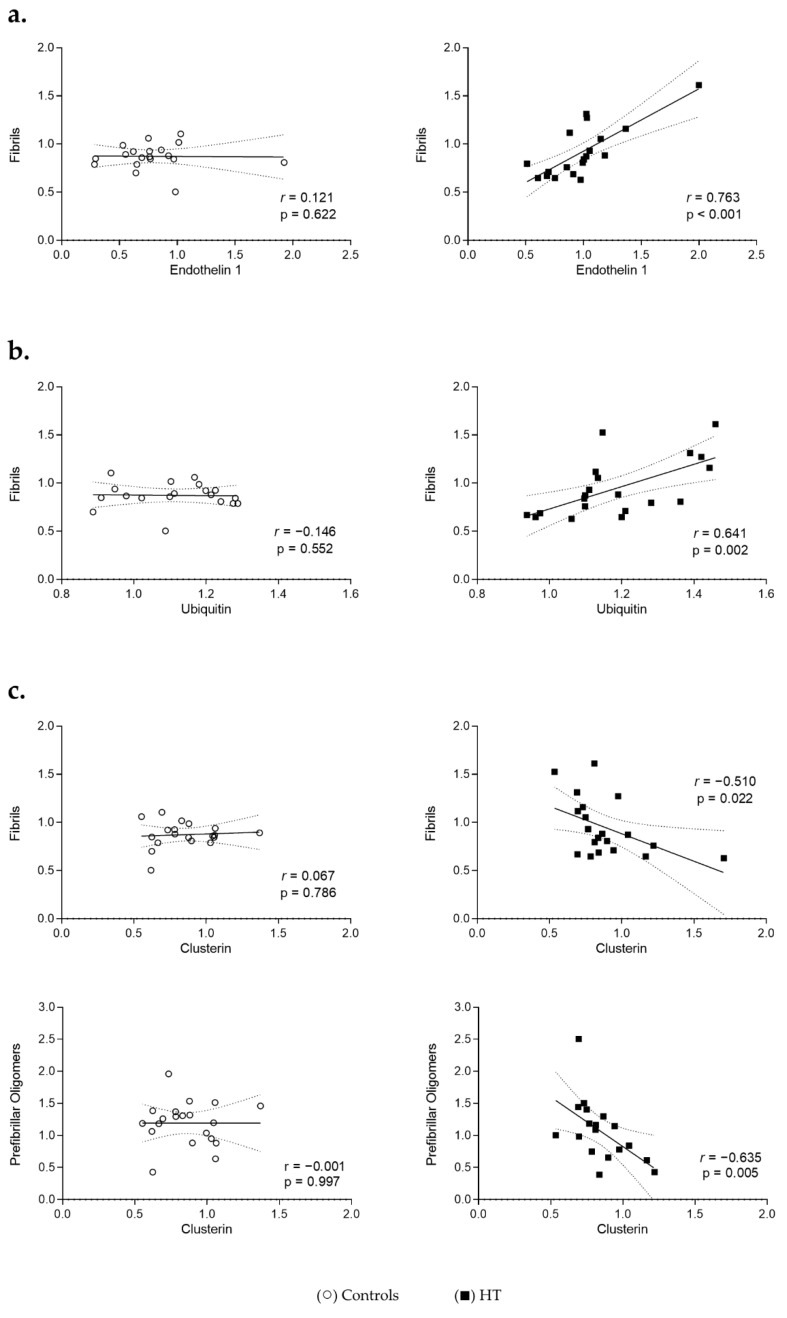
Association of hypertension and proteostasis-related proteins with protein aggregates. Plasma levels of Endothelin 1 (**a**) and Ubiquitin (**b**) and Clusterin (**c**) were matched with the corresponding individual levels of fibrillar or prefibrillar proteins and peptides in controls (○) and individuals with hypertension (■). Spearman’s rank coefficient was applied to test the strength and direction of the relationships between the different variables. The resulting coefficients (r) and corresponding *p*-values (p) are represented. *p* < 0.05 was considered statistically significant. All samples were normalized to the reference sample and protein levels are represented as fold changes relative to this reference sample. Lines represent a linear regression fit with corresponding 95% confidence bands (dotted lines).

Considering the changes observed in the associations of Endothelin 1 with Ubiquitin in HT individuals, the interaction of the proteostasis components with the different forms of aggregated proteins in plasma was further analysed. Different profiles were observed for HT and control individuals. In the HT group, higher levels of plasma Endothelin 1 or Ubiquitin were strongly and significantly associated with higher amounts of fibrils (r = 0.763, *p* < 0.001 and r = 0.641, *p* < 0.01, respectively) (Figure 4a,b). In control samples, these associations are absent (r = 0.121, *p* = 0.62 for Endothelin 1; r = −0.146, *p* = 0.55 for Ubiquitin). Regarding Clusterin, higher levels of this chaperone were significantly associated with decreased fibril levels (r = −0.510, *p* = 0.02) in HT individuals but not in control ones (r = 0.067, *p* = 0.79) (Figure 4c, upper graph). A similar strong negative association was observed between the plasma levels of Clusterin and the prefibrillar oligomer ones in HT samples (r = −0.635, *p* < 0.01), but again not in controls (r = −0.001, *p* = 1.00) (Figure 4c).

## 4. Discussion

From a public health perspective, regulating BP values is a priority to revert the heavy burden associated with hypertension and subsequent cardiovascular diseases [4,30]. However, the molecular networking involved in hypertension is still poorly understood and hard to manage due to the complexity that arises from the many genes, proteins, and metabolites that interact to regulate multiple systems involved in its pathophysiology. These include inflammation, immune response, oxidative stress, vasoactive substances, and proteostasis [8,16].

Failure of the proteostasis network is associated with ageing and several pathological conditions, including neurodegenerative diseases and cardiac disorders [21,31]. Indeed, molecular chaperones, autophagy, and the ubiquitin–proteasome system (UPS) are all essential for maintaining proper cardiac function [16]. In hypertension, the constant mechanical and metabolic aggression of the heart and endothelial cells, caused by high BP, may have the capacity to affect the folding and integrity of proteins [17]. Evidence of proteostasis imbalance in hypertension is being delivered by pre-clinical studies [32,33,34], but few clinical studies have addressed this issue due to difficulties in sampling [27]. Although challenging, exploring protein aggregation in human peripheral fluids can be a promising tool to uncover the role of imbalanced proteostasis in both ageing and pathological conditions [35,36]. The present study evaluated protein aggregation and the dynamics of proteome stability in plasma samples from individuals with hypertension, to investigate the interaction between hypertension and proteostasis.

Protein aggregates can form intracellular inclusions or be secreted from the original cells, or expelled into circulation, through cell death or extracellular vesicles, eventually being eliminated or deposited in distant tissues [37]. To assess and compare the levels of protein aggregation in individuals with hypertension and age- and gender-matched controls without hypertension, plasma samples were first stained with two distinct fluorescent probes. Total protein aggregation assessed either with Proteostat or ThT revealed similar levels in both groups of individuals. For this reason, we focused our analysis on the structural nature of those aggregates. The aggregation of misfolded proteins or peptides can proceed from early aggregates of highly unstructured, partially folded, or native-like species, towards thermodynamically favourable and highly structured assemblies [22,38]. In this process, early aggregates undergo structural reorganizations to produce prefibrillar aggregates, often designated oligomers, which can further aggregate into fibrillar structures [22]. In protein aggregation diseases, both prefibrillar oligomers and fibrillar aggregates exhibit pathogenic properties by inducing cellular damage or compromising organ integrity. Of note, the aggregates found in CVD are structurally similar to those found in neurodegenerative diseases and are able to form amyloid structures [16,39]. The aggregation status of proteins and peptides present in the plasma of individuals with hypertension and normotensive controls was here evaluated with conformation-specific and sequence-independent antibodies that detect soluble prefibrillar oligomers [40] or mature fibrils [41]. Once again, no obvious differences were observed in the levels of prefibrillar oligomers or fibrillar structures between both groups. FTIR analysis confirms the structural similarities between the plasma proteomes of individuals with or without hypertension. All these data suggest that: (i) hypertension, per se, does not increase the levels of these protein aggregates in human plasma; or that (ii) the levels of these plasmatic protein aggregates are relatively controlled in medicated individuals diagnosed with hypertension.

Favouring our second hypothesis, different studies propose that hypertension has an impact on protein aggregation and that the aetiology or pathophysiology of this condition might be associated with unpaired proteostasis [17,34,42]. It is possible, however, that the human organism has the ability to cope with protein aggregation promoted by elevated blood pressure at earlier and/or controlled stages of hypertension. If this hypothesis is true, a new balance would be achieved between the formation of new protein aggregates and their elimination by the proteostasis network. To test it, we analysed the levels of proteins relevant for both hypertension (Endothelin 1) and proteostasis (Ubiquitin and Clusterin), and their putative associations with the different forms of protein aggregates (oligomers and fibrils). Despite having their blood pressure under control, the hypertension group showed higher levels of Endothelin 1 in comparison to the reference group. Endothelin 1 is a well-known potent vasoconstrictor that is highly associated with endothelial dysfunction [43], and similar results have been previously reported [11]. Body mass index was also higher in the hypertension group and presented significant associations with Endothelin 1 in the whole study population (*p* = 0.03). This positive association with Endothelin 1 is not surprising, since excessive weight is a major cause of hypertension, and associations between Endothelin 1 and obesity have been reported [44,45].

Protein quality control mechanisms, like the proteasome, are also associated with Endothelin 1 and other vasoconstrictors [46,47]. In this work, we analysed the levels of the small regulatory protein Ubiquitin and the extracellular chaperone Clusterin in human plasma, as well as their association with Endothelin 1. Ubiquitin tags aggregated or aggregation-prone proteins for elimination through the proteasome or autophagy [48,49]. The UPS tries to dispose of protein aggregates at early stages while proteins that resist this dismantlement can be cleared by autophagy. Clusterin acts as an ATP-independent molecular chaperone by binding to hydrophobic regions of misfolded proteins and preventing protein aggregation [50]. Importantly, Clusterin levels increase in response to CVD-associated stress conditions [51]. Our data show that plasma levels of total Ubiquitin and Clusterin were similar in both groups, but reveal a positive significant association between Endothelin 1 and Ubiquitin in the plasma of individuals with hypertension. This association was not observed in the control group, which suggests a specific activation of the early ubiquitin protein quality control mechanism to the increased production of Endothelin 1 and hypertension.

**Figure 5:**
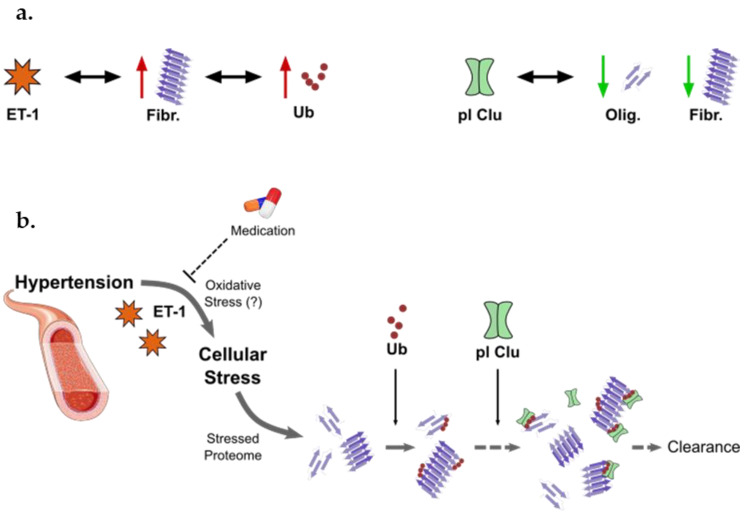
Proposed proteostasis response in individuals with controlled hypertension. (**a**) Associations found between the levels of Endothelin 1 (ET-1), the proteostasis-associated proteins Ubiquitin (Ub) and plasma Clusterin (pl Clu), and Oligomers (Olig.) and Fibrils (Fibr.), in the hypertension group. (**b**) The proteostasis response to hypertension here proposed involves the subsequential action of Ubiquitin and Clusterin as a coping mechanism to restore proteome balance. In this, Ubiquitin first tags oligomers and fibrils that continuously arise from endothelial dysfunction, and these tagged aggregates are subsequently targeted by the plasma chaperone Clusterin, to prevent further aggregation and promote their clearance. (Image source for blood vessel: Servier Medical Art, https://smart.servier.com/ (accessed on 7 January 2022)).

We further explored putative associations of Endothelin 1 and the proteostasis network with the different forms of aggregates found in plasma. Our results show that both Endothelin 1 and Ubiquitin are positively associated with fibrillar proteins and peptides. In line with this, amyloid beta (Aβ) oligomers were shown to increase the levels of reactive oxygen species that subsequently trigger the release of Endothelin 1 leading to reduced cerebral blood flow in Alzheimer’s disease [52]. We also observe that higher Clusterin levels are correlated to lower levels of both fibrils and prefibrillar oligomers. Importantly, all these strong (r > 0.5) associations are specifically observed in plasma of individuals with controlled hypertension and completely absent in normotensive controls. These observations suggest that in a normal health condition proteostasis is well-balanced and that the pathophysiology of hypertension may involve a disruption in protein quality control. Further, chronic activation of the proteostasis mechanisms may cause their overload and failure [23,53]. In hypertension, the constant aggression of the high BP on the cardiovascular system may chronically activate the UPS, halting and damaging its normal activity. Together, the data here presented, support a mechanism where proteostasis response against hypertension-associated protein aggregation involves an earlier step of Ubiquitin-tagging of the protein aggregates, and subsequent action of Clusterin to resolve these functionally impaired tagged proteins in human plasma (Figure 5). Scheidt et al., demonstrated that increasing concentrations of Clusterin progressively reduced protein aggregation by suppressing the rate of elongation of the Aβ (M1-42) fibrils [54]. On the other hand, Turkieh et al. showed that Clusterin levels are increased in plasma and left ventricle after myocardial infarction and that such increase is associated with alterations in the proteasome and autophagy [55,56]. Supporting our hypothesis of a Ubiquitin–Clusterin dual response (Figure 5), Zoubeidi et al. showed that intracellular Clusterin can interact with Ubiquitin to promote the degradation of target proteins through the UPS and that the secreted form of Clusterin also aids the proteasome activity [51].

There are some limitations to be acknowledged in this study and future perspectives to address. Most of the individuals with hypertension recruited in this study were at a mild stage and had well-controlled BP, as evidenced by the similar systolic and diastolic BP levels between both groups. This fact probably overshadowed proteostasis alterations associated with hypertension. Indeed, the medication itself may have a direct disaggregation effect on protein aggregates [57,58], and some of the variables here analysed may vary with the stage of the disease, as well [29,58]. Regarding the different drug classes represented in the studied population, Statins were already shown to interfere with protein quality control [59,60]. However, this class of drugs is similarly present in both HT and Control groups and should not interfere with our findings. Antihypertensive drugs have also been reported to mask some cardiovascular biomarkers and were linked with changes in proteostasis and protein aggregation [61]. For instance, Malik et al. [62] showed that diuretics can interfere with in vitro formation of amyloid structures, while Cicalese [63] and collaborators reported that Angiotensin II is connected to proteotoxicity in vascular smooth muscle cells and that the treatment with a chemical chaperone attenuated those effects. ARBs can interact with Angiotensin II-mediated signalling, possibly changing protein quality control responses. Altogether, a parallel impact of the different drug classes or combined therapies in proteostasis should not be excluded, although we did not find any significant associations between the intakes or not of distinct drug classes and the studied protein aggregation markers.

The number of participants in this study is also a limitation, and not enough to investigate the effect of the different drug classes on protein aggregation and proteostasis. Further investigation with a higher number of individuals is important to clarify and strengthen these findings. The analysis of protein aggregates should also be applied to plasma samples of patients with a more aggressive prognostic, uncontrolled hypertension, resistant and refractory hypertension, or even an established CVD like heart failure, coronary heart disease, or atherosclerosis, in which alterations in protein conformations and quality control mechanisms could be greater than those observed in mild hypertensive individuals. For example, a study from Gouveia et al. revealed that the plasma proteome of heart failure preserved injection fraction patients is characterized by the presence of secondary structures often found in protein aggregates [64]. Although the main goal of this study was to evaluate protein aggregation in individuals with hypertension and its association with the proteostasis network using a minimally invasive procedure, it would also be relevant to validate our findings in cardiomyocytes or endothelial cells. Finally, it would also be interesting to study the effect of non-pharmacological therapies, such as exercise, on these proteostasis biomarkers. Physically active hypertension patients, for example, had less plasma Ubiquitin and higher levels of its associated HSP70 than a sedentary age-matched group with hypertension [65].

## 5. Conclusions

Although no obvious change was detected in the plasmatic protein aggregation profile of participants with mild and controlled hypertension, the associations described in this work evidence that protein quality control mechanisms and protein aggregation may be both affected and interlinked in the pathophysiology of hypertension. Despite their well-controlled BP, changes in proteostasis caused by a frail haemodynamic remain in the background in individuals diagnosed with and medicated for hypertension. Overall, it seems that the human body has the capacity to cope with these changes in proteostasis during mild, controlled, stages of hypertension, potentially through an interlinked action of Ubiquitin and Clusterin. However, chronic activation of these proteostasis mechanisms may lead to their overload and failure, contributing to the progression of hypertension and associated diseases. Studying protein quality control systems in individuals just diagnosed with hypertension or affected with associated CVDs could provide valuable information in the characterization of diseases state and reveal new therapeutic targets that could drastically change the outcome of such conditions.

## Figures and Tables

**Table 1:** General characteristics and medication of the participants.

	Control Group	HT Group	
Characteristics, Mean ± SD	*n* = 20	*n* = 20	*p* Value
Male/Female (*n*)	8/12	5/15	0.324
Age (years)	64.8 ± 6.4	65.6 ± 6.1	0.750
BMI (kg/m^2^)	26.1 ± 3.2	30.3 ± 5.8	0.009
Waist Circumference (cm)	92.4 ± 11.6	104.2 ± 13.1	0.006
Office SBP (mm Hg)	126.1 ± 11.2	126.0 ± 14.1	0.979
Office DBP (mm Hg)	73.7 ± 7.8	71.5 ± 8.1	0.407
HR (bpm)	65.6 ± 8.5	66.2 ± 10.8	0.840
**Medical history**	**No. (%)**	**No. (%)**	***p*** ** value**
Diabetes	1 (5)	4 (20)	0.341
Obesity	3 (12)	10 (50)	0.041
Overweight	11 (55)	6 (30)	0.333
Dyslipidaemia	11 (55)	16 (80)	0.176
**Medication**	**No. (%)**	**No. (%)**	***p*** ** value**
ACEI	-	3 (15)	0.231
ARB	-	10 (50)	<0.001
Diuretics	-	13 (65)	<0.001
CCB	-	4 (20)	0.106
Β-Blockers	-	3 (15)	0.231
Statins	8 (40)	10 (50)	0.751

Data given as ‘mean ± SD’, unless otherwise indicated. ACEI: Angiotensin-converting-enzyme inhibitor; ARB: Angiotensin II receptor blocker; BMI: body mass index; CCB: Calcium channel blocker; DBP: diastolic blood pressure; HR: heart rate; SBP: systolic blood pressure.

## Data Availability

The datasets generated and analysed during the current study are not publicly available since it is study performed with samples collected from volunteers, and therefore containing personal parameters, but are available from the corresponding author on reasonable request.

## Supplementary Materials

The following supporting information can be downloaded at: https://www.mdpi.com/article/10.3390/cells11101686/s1, Figure S1: FTIR spectra of human plasma of individuals with hypertension (black) and controls (grey). (a) Overview of baseline-corrected, normalized FTIR spectra of hypertension (HT) and control groups in the region 4000–900 cm^−1^. (b) Amplification of the amide I region (1700–1600 cm^−1^) (left) and 2nd derivative of spectra of amide I region, using Savitzky-Golay algorithm with 3 smoothing points (right).
