# Supplementary figures and images for: Proteostasis Response to Protein Misfolding in Controlled Hypertension
Source: Cells. 2022 May 19;11(10):1686. doi: 10.3390/cells11101686 (PMC9139827; doi:10.3390/cells11101686)

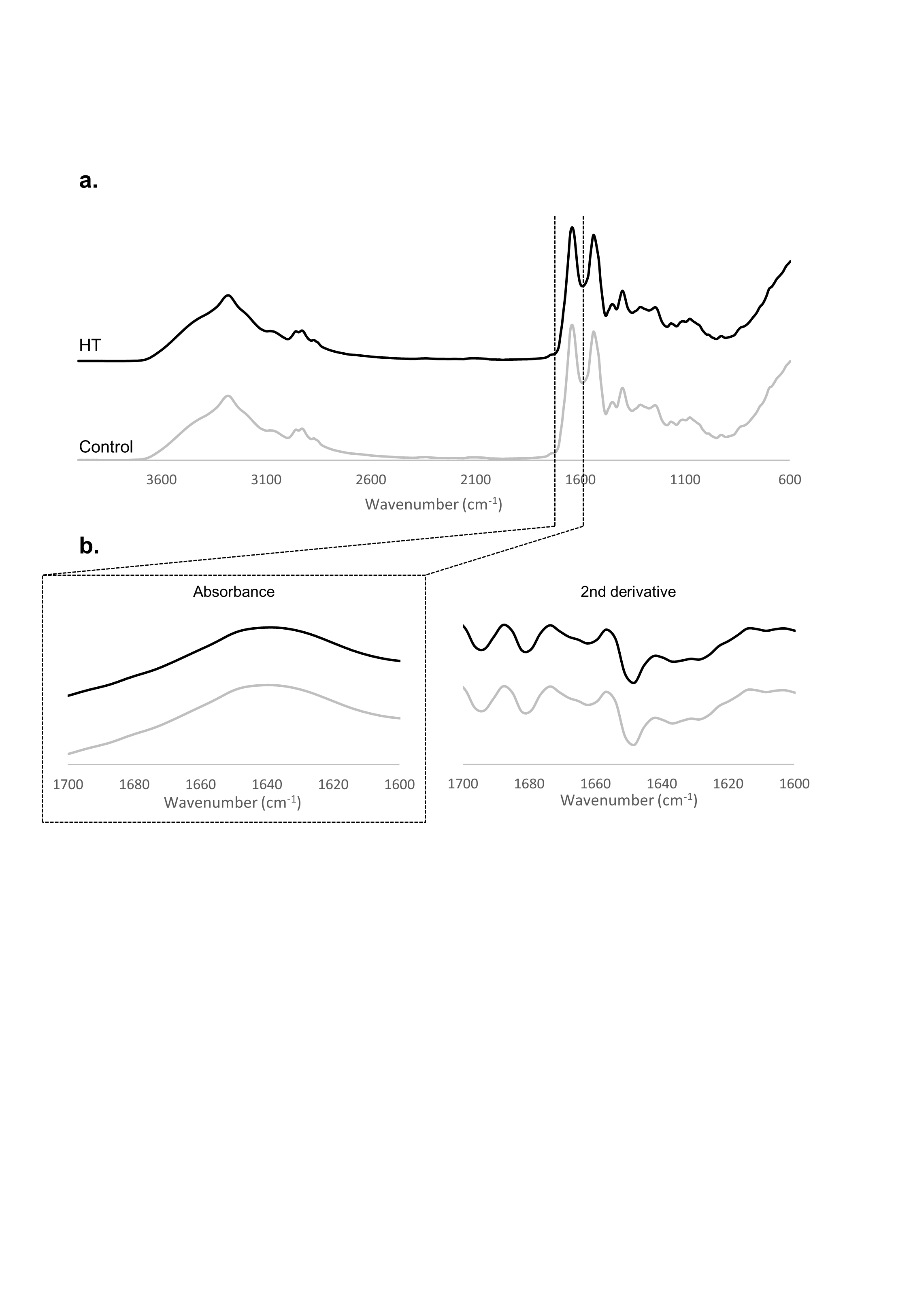

Supplement: Supplementary file 1 [file cells-11-01686-s001.zip › cells-1675361-supplementary.tif]
